# The challenges of finding the gene responsible for a rare, autosomal dominant gastric cancer susceptibility syndrome

**DOI:** 10.1186/1897-4287-10-S2-A71

**Published:** 2012-04-12

**Authors:** JJ Li, S Healey, K Phillips, I Makunin, N Wayte, I Schrader, D Worthley, N Lindor, D Huntsman, D Goldgar, G Suthers, G Chenevix-Trench

**Affiliations:** 1 Queensland Institute of Medical Research, Australia; 2 South Australian Clinical Genetics Service, Australia; 3 Hereditary Cancer Program, BC Cancer Agency, BC, Canada; 4 Division of Digestive and Liver Diseases, Columbia University, NY, USA; 5 Department of Medical Genetics, Mayo Clinic College of Medicine, MN, USA; 6 Department of Dermatology, University of Utah School of Medicine, Salt Lake City, USA

## 

Gastric Adenocarcinoma and Proximal Polyposis of the Stomach (GAPPS) is a newly described, rare autosomal dominant syndrome, characterized by fundic gland polyposis with occasional hyperplastic and adenomatous polyps. We diagnosed GAPPS in a large Australian (Family 1) and two smaller North American families (Family 2 and 3). Mutations in *APC*, *MUTYH*, *CDH1*, *SMAD4*, *BMPR1A*, *STK11* and *PTEN* were excluded in all families by sequence analysis of exons and flanking regions, as well as by assays for deletion or duplication of exons. We mapped the GAPPS gene in Family 1 by linkage analysis (LOD score 4.21) to a 20Mb region which contains about 60 genes. Short tandem repeat genotyping showed that the affected members of Family 2 share a haplotype in this 20Mb region, but analysis of Affymetrix SNP 6.0 data from three affected members of Family 2 showed that they also shared several other regions of the genome. However, both Family 2 and 3 are too small for definitive linkage analysis.

We have carried out full exome sequencing for three affected individuals in Family 1 (and targeted sequencing of the linked region in another individual), and of three affected members of Family 2. We did not find any rare coding or splice site variants in the linked region that were shared by all affected members of Family 1; nor any from the same region in both affected members of Family 2. All coding exons and miR genes in the linked region have been sequenced at least 30X at every base, or by Sanger sequencing, except for seven exons for which Sanger sequencing is yet to be completed. We will also use Sanger sequencing to improve the coverage of exons in the linked region in Family 2.

However, our current hypothesis is that non-coding mutations in the linked region are responsible for the GAPPS syndrome in both Families 1 and 2 and so whole genome sequence analysis for two affected members in Family 1 has been performed and analysis is underway.

